# The effects of sport expertise and shot results on basketball players’ action anticipation

**DOI:** 10.1371/journal.pone.0227521

**Published:** 2020-01-06

**Authors:** Yawei Li, Tian Feng

**Affiliations:** 1 Department of Sports, Physical Education College of Zhengzhou University, Zhengzhou, China; 2 Department of Social Sports, Physical Education College of Zhengzhou University, Zhengzhou, China; University of Texas at San Antonio, UNITED STATES

## Abstract

The purpose of the present cross-sectional study was to clarify the effects of sport expertise and shot results on the action anticipation of basketball players. Eighty-eight male subjects participated in this study, namely, 30 collegiate basketball players, 28 recreational basketball players and 30 non-athletes. Each participant performed a shot anticipation task in which he watched the shooting phase, rising phase, high point and falling phase of a free throw and predicted the fate of the ball. The results showed that the collegiate players and recreational players demonstrated higher accuracy than the non-athletes for the falling phase but not for the other temporal conditions. Analysis of the shot results demonstrated that for made shots, the collegiate players and recreational players provided more accurate predictions than the non-athletes. These results suggested that the experienced players required a sufficient amount of information to be able to make accurate judgements and demonstrated that the experts’ judgement bias for made shots was independent of the temporal condition.

## Introduction

Action anticipation refers to the ability to predict the outcome of an event [1]. In contrast to racquet sports (such as tennis or badminton) in which a server usually decides the ball direction before starting his or her action, a basketball shooter can never be certain of the shot result until the final second. Thus, illustrating the development of shot anticipation ability in basketball is critical. Anticipating the outcome of an action, such as predicting whether a basketball will hit or miss the basket, directly affects the performance of an athlete. Accurate judgement helps a player plan his or her subsequent action (such as obtaining a rebound or playing defence) accordingly. Thus, the main goal of this study was to investigate the influence of sport expertise and shot results on the action anticipation of basketball players.

Studies have shown that sport expertise improves action anticipation ability. Aglioti and Cesari [2] asked basketball athletes, spectators (coaches or journalists) and novices to predict the success of free throws and found that the athletes responded earlier and more accurately than the other two groups of participants. Similarly, Uchida and Mizuguchi [3] compared the anticipation performance of experienced basketball players and novices observing a basketball free throw. The results demonstrated that the correct response rate among the experienced players was significantly greater than random chance (50%) and that of the novices, while the novices’ correct response rates were close to random chance. The distinct visual strategies used by athletes and novices may account for the observed differences. The researchers found that the basketball players focused more on body cues (i.e., the shooter’s knee, wrist and finger joint angles), while the novice group focused more on the ball [2, 3].

Temporal information has been confirmed to play a necessary role in predicting outcomes, and the advantage of experts has been revealed in early time phases. Wu and Zeng [4] divided the time course of a free throw into three different phases with 11 sequential pictures as follows: the basketball leaving the model player’s hand (3 pictures), the basketball reaching the climax of its trajectory (6 pictures), and the basketball approaching the basket (9 pictures). The participants were required to predict the ball’s fate based on varying temporal information under different temporal conditions. The researchers found that when the participants observed 3 or 6 pictures, the athletes achieved higher prediction accuracy than the novices, indicating that experts utilize early cues better than novices when making predictions without complete information. Consistently, studies involving tennis or table tennis have shown that regional-level athletes use early valid information and achieve higher prediction accuracy than college-level and novice groups [5, 6].

The result of a shot may be related to the anticipation advantage of elites, although most previous studies have concentrated only on the results of anticipation (i.e., correct or incorrect). The interaction between the actual results and anticipation yields the following four situations: true positive (correct prediction that a shot would be successful), false positive (incorrect prediction that a shot would be successful), true negative (correct prediction that a shot would be unsuccessful), and false negative (incorrect prediction that a shot was unsuccessful). Some studies have sought to determine the effect of shot results on anticipation at different visual angles. Cañal-Bruland and Balch [7] asked basketball players and observers to perform and observe shots. Both the players’ and observers’ vision was occluded at the time of the ball’s release, and they were asked to predict the ball’s fate. The authors found that the players were better at judging their own shots as “in” than judging others’ shots. To the best of our knowledge, only subjects who lack experience may have worse prediction accuracy than random chance (50%). Interestingly, a recent study by Maglott and Chiasson [8] found that for missed shots, collegiate shooters had worse prediction accuracy for their own shots than recreational shooters, and their prediction accuracy was significantly worse than random chance. Moreover, signal detection theory implies that collegiate players show a higher bias towards predicting that the shot result is “in”.

This judgement bias seems to be caused by the presence of the "regulatory fit" effect [9]. According to the theory of regulatory focus, two types of individual focus exist, namely, promotion focus and prevention focus [9]. Individuals with a promotion focus are eager to succeed and adopt more positive behavioural strategies, while individuals with a prevention focus are more inclined to avoid failure and adopt conservative behavioural strategies [10]. The effect emerges when the action strategy is consistent with one’s focus. Moreover, research conducted by Memmert and Unkelbach [11] shows that three-point shooting in basketball is more likely to be a task involving a promotion focus, and athletes exhibit better behavioural performance when matching with the action strategy. Therefore, predicting a shot as “in” is a positive strategy that may fit experts’ promotion focus, cause a regulatory fit, and thus improve their accuracy. In contrast, novices are more likely to have a prevention focus and exhibit superior performance when the ball is out.

Concerning the influence of the shot result, some important questions have been raised, but the answers remain unclear. Notably, experts and novices show different anticipation abilities in predicting the shot results of “in” and “out”. Will athletes exhibit an advantage in predicting successful shots when the shots are divided into varying time phases? Will the performance of athletes and novices change under different conditions of temporal occlusion? We sought to answer these questions by evaluating three groups of individuals with varying levels of basketball expertise (collegiate players, recreational players and non-athletes) and investigating the effect of shot results on action anticipation in different time phases. Both recreational players and non-athletes were included to determine whether a certain amount of expertise may confer a prediction advantage. We hypothesized that (1) compared to the non-athletes, the collegiate players and recreational players will show superior anticipation performance under the early temporal condition(s) and that the collegiate players will outperform the recreational players under the early temporal condition(s); (2) under the early temporal condition(s) of made shots, both collegiate players and recreational players will perform better than the non-athletes and that the collegiate players will be more accurate than the recreational players; and (3) for missed shots, the two experienced groups will provide more accurate predictions than the non-athletes under the early temporal conditions and that the collegiate players will outperform the recreational players.

## Materials and methods

### Ethical approval

This study was carried out ethically and was approved by the Ethical Committee of Physical Education College of Zhengzhou University (No. 2019001). The individual in this manuscript has given written informed consent (as outlined in PLOS consent form) to publish these case details.

### Participants

Eight-eight male subjects participated in this study, including 30 collegiate players (age: 21.53±0.97 years, height: 1.87±0.06 m, mass: 91.3±16.9 kg), 28 recreational players (age: 21.14±0.85 years, height: 1.79±0.04 m, mass: 77.6±14.7 kg) and 30 non-athletes (age: 21.00±1.15 years, height: 1.74±0.06 m, mass: 64.9±9.9 kg). The collegiate players participated on the basketball team of the Physical Education College of Zhengzhou University and practised 10.61±2.40 hours per week. The recreational players were collegiate track and field, boxing, rowing and judo athletes. These players participated in basketball games recreationally for 3.20±1.55 hours per week. The non-athletes were university students who had never participated in any sports training. The three groups did not vary in age, *F*(2, 83) = 2.23, *p* = 0.11, η_p_^2^ = 0.51. Informed consent regarding the purpose and methods of the study and the obligations, responsibilities and rights of the subjects was obtained prior to the experiment.

### Materials

Notably, we interrupted the complete sequence of each throw at one of four possible clip durations. We used continuous pictures rather than a video for the experimental stimuli since the selected pictures provided important information (for anticipation of the ball being “in” or “out”) that was more stable than information provided by a video. Two professional right-handed male athletes were required to shoot 60 free throws after warming up. All shots were recorded by a digital camera (Canon EOS 5D Mark IV, focal length of 3.5 mm). The camera height was 1.70 metres. Each shot was recorded at a speed of 6 frames per second from when the player held the ball to when the ball hit (or missed) the basket. Finally, at total of 40 free throws were selected according to the flight phase of the ball, including 20 made shots and 20 missed shots.

**Fig 1:**
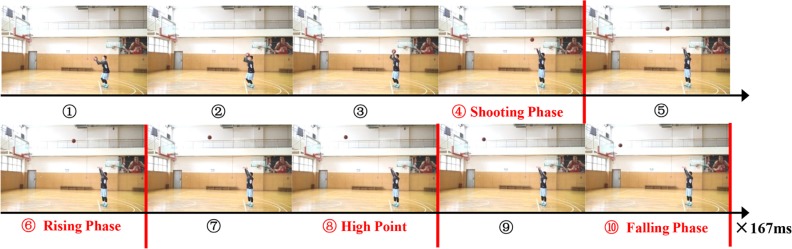
Example of the experimental stimuli. Continuous pictures of free-throw shooting as shown to the subjects. The pictures depict the time from when the player held the ball to the time when the ball hit (or missed) the basket. Each picture is presented for 167 ms, and the red line depicts when the shot stops under the 4 conditions of the temporal experimental stimuli.

According to temporal information, the flight phase of a basketball and previous research results, each shot was divided into the following four temporal conditions: (1) the shooting phase, (2) the rising phase, (3) the high point, and (4) the falling phase. The final two pictures (pictures 11 and 12) were excluded from the experimental stimulus set to prevent the subjects from seeing the shot results. The exposure time for each picture was 167 ms, which was the same as the time required to take the pictures (6 pictures per second). Table 1 shows the characteristics of each temporal condition (the number of pictures, presentation time and ball position), and Fig 1 presents an example of the experimental stimuli.

**Table 1:** Experimental stimulus information.

Temporal Condition	Number of Pictures	Presentation Time (ms)	Ball Position
(1) Shooting Phase	4	668	The player releases the basketball.
(2) Rising Phase	6	1002	The basketball reaches approximately the midpoint between the player’s hand and the high point.
(3) High Point	8	1336	The basketball reaches the climax of its trajectory.
(4) Falling Phase	10	1670	The basketball approaches the basket.

### Procedures

The experiment was conducted in a quiet room at the Physical Education College of Zhengzhou University, and each participant was tested individually under the supervision of an experimenter. The participants completed a questionnaire regarding their individual information (i.e., sex, age, training year, height, mass, and training hours per week) and then participated in an action anticipation task. In the experiment, the participants were seated in front of a screen (23.8 inches) at a distance of 60 cm. A two-alternative forced choice task requiring the participants to predict the result of a free throw was conducted. The task began with a black cross-shaped fixation point on a white background displayed at the centre of the screen for 2,000 ms. Then, continuous pictures of a free throw were displayed, and each picture was presented for 167 ms at a resolution of 1024 x 682 pixels. After viewing all photos within each trial, the participants predicted the fate of the ball (made or missed by pressing the “F” key or the “J” key, respectively) quickly with the goal of being accurate. Responses submitted more than 3,000 ms after presentation of the stimulus were considered errors. The subsequent trial started immediately following completion of the previous trial. After reading the standardized task instructions, the subjects were asked to complete 15 practice trials with feedback (including 4, 6, 8 and 10 pictures). The pictures used in the practice trials were not used in the formal experiment. If a participant’s accuracy during the practice trials did not exceed 60%, i.e., the subject made more than 6 incorrect judgements, then he did not pass the practice session and was required to perform an additional practice session. Two collegiate players, two recreational players and five non-athletes required two practice sessions to meet the requirement. One collegiate player and one non-athlete required three practice sessions to meet the requirement. The experiment included 160 trials (40 shots×4 temporal conditions, 668 ms, 1002 ms, 1336 ms and 1670 ms), and the 40 shots included 20 successful shots and 20 missed shots under each temporal condition. All trials were divided into 8 blocks, and each block included 20 shots (both successful and missed) under one of the four conditions. The experiment was designed and displayed with E-prime 2.0 (Psychology Software Tools, Inc., Pennsylvania, USA). The response keys for the made and missed shots were counterbalanced across the subjects, and no feedback was provided during the experimental trials. A 30-second break was provided between each block. The entire experiment lasted approximately 50 min.

### Statistical analyses

Assessing the reaction time of the participants is difficult because the participants were able to make their decisions before the option to press the key became available, and each prediction was made after the picture disappeared. Therefore, only the accuracy results were evaluated. The accuracies in the three groups under each temporal condition exhibited normal distributions (*z* < .803, *p*>.539 in all instances). To test Hypotheses 1, 2 and 3 regarding the performance of the three groups under different temporal conditions and shot results, a mixed-design three-way analysis of variance (ANOVA) model with accuracy as the dependent variable, group (collegiate players, recreational players, and non-athletes) as the between-subjects factor, and temporal condition (668 ms, 1002 ms, 1336 ms and 1670 ms) and shot result (made shots and missed shots) as the within-subjects factors was calculated. Greenhouse-Geisser correction was applied when the assumption of sphericity was violated, and Bonferroni-corrected post hoc *t*-tests were used to identify the main effects and interactions.

## Results

**Fig 2:**
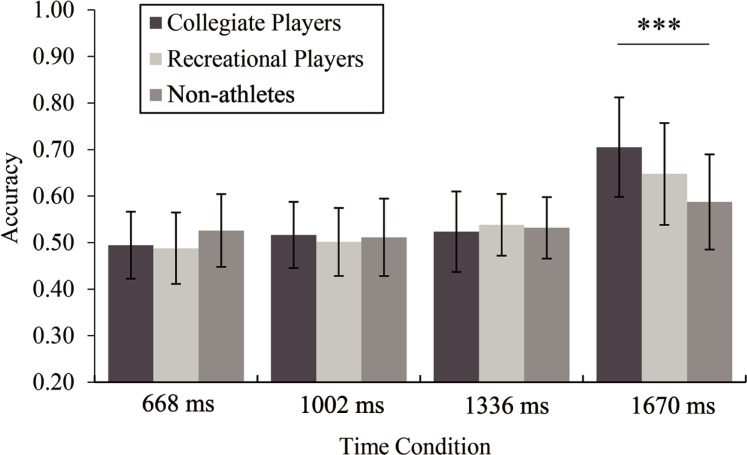
Accuracy (mean±*SD*) under each temporal condition per group.

The ANOVA of accuracy indicated significant main effects of the temporal condition, *F*(3, 255) = 65.20, *p*<0.001, η_p_^2^ = 0.43, and shot result, *F*(1,85) = 59.15, *p*<0.001, η_p_^2^ = 0.41, but not group, *F*(2, 85) = 1.40, *p* = 0.253, η_p_^2^ = 0.03. The interaction between the temporal condition and group was significant, *F*(6, 255) = 5.69, *p*<0.001, η_p_^2^ = 0.12. The post hoc tests of accuracy under the four temporal conditions per group revealed that under the 1670-ms condition, the collegiate players (*M* = 0.71, *SD* = 0.11) showed better accuracy than the non-athletes (*M* = 0.59, *SD* = 0.10, *p*<0.001), but the recreational players (*M* = 0.65, *SD* = 0.11) did not differ from the other two groups of participants (*p*>0.112 in all instances). No significant difference was found among the three groups under any other temporal conditions (*p*>0.173 in all instances, see Fig 2.).

**Fig 3:**
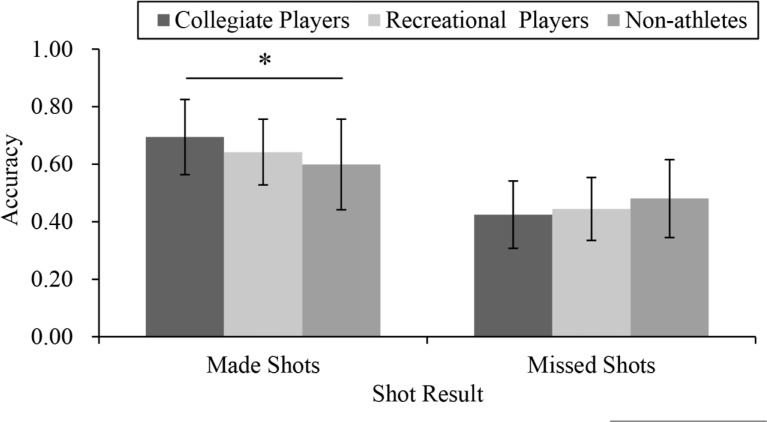
The accuracy (the mean±*SD*) of each shot result per group under all time conditions.

Additionally, a marginally significant interaction was found between the shot result and group, *F*(2, 85) = 3.02, *p* = 0.054, η_p_^2^ = 0.07. Fig 3 shows the results of the post hoc tests. Under the made shot condition, the collegiate players were superior to the non-athletes (collegiate players: *M* = 0.69, *SD* = 0.13, non-athletes: *M* = 0.60, *SD* = 0.16, *p*<0.05), but the recreational players (*M* = 0.64, *SD* = 0.11) did not differ from the other two groups of participants (*p*>0.440 in all instances). For the missed shot condition, no significant differences between the groups were found (collegiate players: *M* = 0.42, *SD* = 0.12, recreational players: *M* = 0.44, *SD* = 0.11, non-athletes: *M* = 0.48, *SD* = 0.14, *p*>0.243 in all instances).

The interaction between temporal condition and shot result was significant, *F*(3, 255) = 9.54, *p*<0.001, η_p_^2^ = 0.10, and post hoc tests showed that all participants predicted the made shots (*M* = 0.65, *SD* = 0.14) better than the missed shots (*M* = 0.45, *SD* = 0.12) under every temporal condition (*p*<0.001 in all instances). The three-way interaction was not significant, *F*(6, 255) = 0.94, *p* = 0.455, η_p_^2^ = 0.02.

## Discussion

The present study is the first to clarify the effects of sport expertise and shot results on the action anticipation of basketball players. The performance of basketball players with varying skill levels (collegiate players and recreational players) and non-athletes was compared in an action anticipation task. Concerning the temporal conditions and shot results, the results demonstrated that the collegiate players and recreational players exhibited better accuracy than the non-athletes for the falling phase but not for the other temporal conditions. Additionally, all participants showed superior performance for made shots versus missed shots under every temporal condition. Furthermore, for the made shots, the collegiate players demonstrated higher prediction accuracy than the non-athletes. This study is the first to combine different temporal information with shot results to test the anticipation of basketball players with varying skill levels and non-athletes; thus, the results of this study may add important information to the present literature.

In the field of action anticipation, sport expertise and temporal information have attracted considerable interest. Studies focusing on the anticipation of basketball shooting found that when watching early (from 429 ms to 858 ms) and medium-term (from 426 ms to 852 ms) action phases during a shot, athletes provided more responses and predicted the shot result more accurately, but no difference between groups was found when they watched the late action phase of the shots [2, 4]. However, the present study compared the accuracy of basketball players with varying levels of experience and non-athletes under different temporal conditions (0–668 ms, 0–1002 ms, 0–1336 ms and 0–1670 ms after the start of the shot) and demonstrated the advantage of collegiate players under the latest temporal condition (i.e., the phase when the ball approached the basket). This result contradicts the findings of previous studies and Hypothesis 1. This inconsistency may be caused by differences in experimental protocols. Our study required the subjects to make a two-alternative decision regarding whether a shot would be successful or missed, which is similar to actual situations in sports. Accordingly, as the information provided to a subject decreases, the accuracy should approach 50%. The studies conducted by Aglioti and Cesari [2] and Wu and Zeng [4] allowed the subjects to make an uncertain choice (i.e., “I do not know”), which may lead to different response strategies. In the early and medium-term action phases of the shots, the non-athletes were more inclined to choose uncertainty, but the athletes tended to make certain judgements and showed better performance. Furthermore, in the study conducted by Aglioti and Cesari [2], the non-athletes’ accuracy was approximately 80% when watching a shot for 1207 ms (similar to the high point phase in our study), while the present study found that the accuracies of the players and non-athletes were only 52% and 53%, respectively. The differences in the results may be explained by individual differences in the participants. Our results implied that the experienced players required a sufficient amount of information to be able to make accurate judgements.

In addition, other studies concerning anticipation in tennis or table tennis revealed that elite athletes were able to utilize early valid information to facilitate their prediction of the ball direction [5, 6]. Zhao and Lu [5] found that when observing a video of table tennis serves with early kinematic cues and early flight cues, the regional-level group had higher prediction accuracy than the college-level and novice groups. To the best of our knowledge, a tennis server usually determines the ball’s direction before he or she serves. Thus, the server’s body posture presents some directional information that experienced players can use to make accurate judgements. In contrast to tennis serving, basketball shooting is a more uncertain task during which the shooter may think “I hope that the ball goes in, but I am not sure”; thus, early clues may not be present due to the uncertainty of the shot result.

According to previous studies, sport expertise seems to cause an anticipation bias towards made shots. Analysis of the shot results and groups revealed that basketball playing experience selectively influenced the collegiate players’ anticipation of a shot. For the made shots, the accuracy of the collegiate players was significantly higher than that of the non-athletes. Consistent with these results, Cañal-Bruland and Balch [7] and Maglott and Chiasson [8] demonstrated that players shooting a ball were better at judging their own shots as “in” than observers and recreational shooters. Although their experiment required the participants to shoot a ball themselves and then to make a judgement using both visual and proprioceptive information, our study required the participants to judge other people’s shots and showed that the collegiate players performed better than the other groups when presented with made shots, suggesting that experts can match the proprioception of their own shots to the visual information of others’ shots.

Additionally, regulatory focus theory suggests that performance improvement is affected by the "regulatory fit" [12]. Expert basketball players have higher motivation for achievement and must exert their best effort to win a match, conferring a self-regulatory focus, i.e., “focus on gains”, and a behavioural strategy, i.e., “search for a win”. Memmert and Unkelbach [11] found that the regulatory fit led to a broader scope of attention in a basketball shot task. When individuals with a “focus on gains” were required to predict whether positive results would occur (e.g., expert players were required to judge whether a shot would be a successful shot), the regulatory fit could help them improve their performance [13]. Regarding signal detection theory, the goal of “focus on gains” individuals is to ensure a “hit” and prevent a “miss” [12]. Consistent with this theory, the superior performance (e.g., more hits and fewer misses) of the collegiate players in the present study may be due to the effect of the regulatory fit. However, our results were not completely consistent with Hypothesis 2. The present study corroborated that the experts’ accuracy was higher than that of the other groups regardless of the temporal condition, indicating that the anticipation bias of experts existed in all phases of a shot.

By comparing the accuracy of the missed shots, the results showed an interesting trend such that the accuracy of the missed shot predictions gradually decreased as the skill level increased under all conditions. Similarly, Maglott and Chiasson [8] reported that for missed shots, collegiate shooters had poorer prediction accuracy regarding their own shots than recreational shooters, and their prediction accuracy was significantly worse than random chance. Adding a non-athlete group in the present study demonstrated the effect more specifically. The accuracy of the collegiate players and recreational players for missed shots was lower than that of the non-athletes. In addition, the differences between the accuracy in each group and random chance (50%) were compared according to a study conducted by Uchida and Mizuguchi [3]. The accuracy of the collegiate players and recreational players for missed shots was lower than random chance in the first three phases (collegiate players: *t*<-3.65, *p*<0.001 in all instances; recreational players: *t*<-2.26, *p*<0.05 in all instances), but no such difference was observed in the non-athlete group in any shot phase (*t*>-1.13, *p*>0.12 in all instances). Consistent with this finding, studies have found that sometimes skilled athletes’ subconscious can inhibit objective visual information [14]. The subjective expectations of made shots in the present study appeared to represent this type of subconscious, which has also been called overconfidence or desirability bias [7]. Although some researchers may consider this bias a useful tool for experts to successfully shoot a free throw [15], it indeed impeded the experts’ prediction accuracy. At least, the poor performance of the experts reminded the collegiate players to not always think that a shot will be successful.

Some limitations existed in the present study. First, an anticipation error can be corrected by a rebound or reshot if the person who made the judgement realizes his or her mistake. Aglioti and Cesari [2] found that only experts have the ability to distinguish between error and correct judgements. However, the present study did not examine whether the participants could determine whether their judgements were correct or incorrect. Additionally, future studies should determine whether error realization can be affected by temporal information or shot results. Second, the present study required the subjects to respond after viewing 4, 6, 8 and 10 pictures. The experimental protocol was not totally consistent with an actual situation in the sport. In a basketball game, players can predict the ball’s fate at any time after the ball is released; thus, future designs of the anticipation task may use complete videos and allow subjects to make predictions at any time during the video. Finally, the recreational group in the present study included athletes from other sports, such as track and field, boxing, rowing and judo, and whether experience in other sports may transfer to action anticipation is unknown. Therefore, more studies are needed to compare players with different levels of basketball expertise only.

## Conclusions

The present study considered the effects of sport expertise and shot results on the action anticipation of basketball players. When free throws were divided into four temporal conditions (i.e., the shooting phase, the rising phase, the high point and the falling phase), the collegiate players and recreational players demonstrated better accuracy than the non-athletes for the falling phase but not for the other temporal conditions. Concerning the accuracy of the shot results, the results showed that the collegiate players and recreational players had more accurate predictions than the non-athletes when judging made shots in the late time phase. Moreover, an analysis of the missed shots revealed worse performance by the collegiate players compared with the non-athletes in the middle time phase.

## Supporting information

S1 File Raw data. (SAV)

S2 File Ethical approval. (JPG)
