# Supplementary figures and images for: The effects of sport expertise and shot results on basketball players’ action anticipation
Source: PLoS One. 2020 Jan 6;15(1):e0227521. doi: 10.1371/journal.pone.0227521 (PMC6944359; doi:10.1371/journal.pone.0227521)

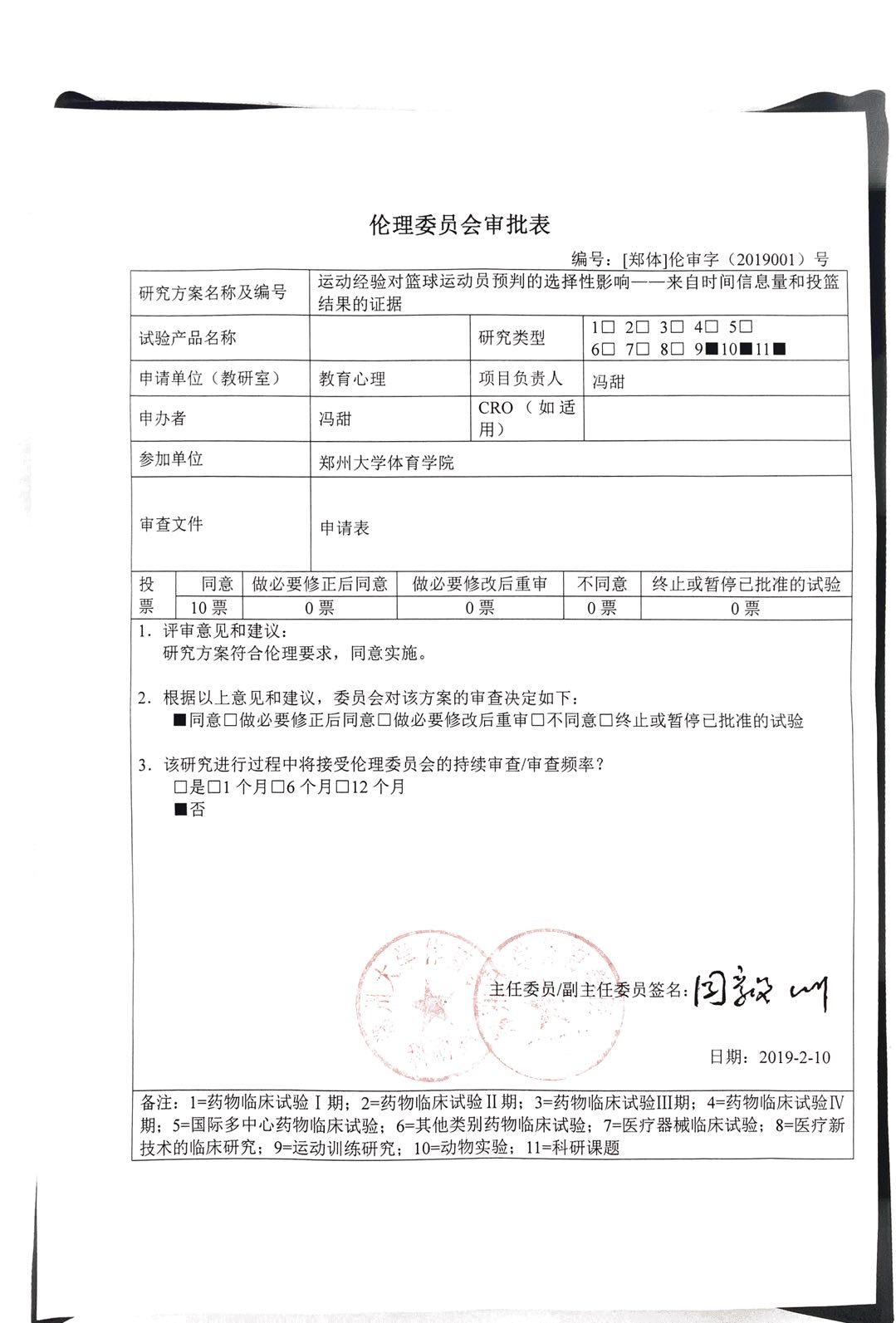

Supplement: S2 File — (JPG) [file pone.0227521.s002.jpg]
